# Beneficial Plant-Associated Microorganisms From Semiarid Regions and Seasonally Dry Environments: A Review

**DOI:** 10.3389/fmicb.2020.553223

**Published:** 2021-01-15

**Authors:** Maria Leticia Bonatelli, Gileno Vieira Lacerda-Júnior, Fábio Bueno dos Reis Junior, Paulo Ivan Fernandes-Júnior, Itamar Soares Melo, Maria Carolina Quecine

**Affiliations:** ^1^Department of Genetics, Luiz de Queiroz College of Agriculture, University of São Paulo, Piracicaba, Brazil; ^2^Brazilian Agricultural Research Corporation, Embrapa Meio Ambiente, Jaguariúna, Brazil; ^3^Brazilian Agricultural Research Corporation, Embrapa Cerrados, Planaltina, Brazil; ^4^Brazilian Agricultural Research Corporation, Embrapa Semiárido, Petrolina, Brazil

**Keywords:** semiarid, seasonally dry environments, plant growth-promoting bacteria, drought, salinity

## Abstract

Semiarid regions are apparently low biodiversity environments; however, these environments may host a phylogenetically diverse microbial community associated with plants. Their microbial inhabitants are often recruited to withstand stressful settings and improve plant growth under harsh conditions. Thus, plant-associated microorganisms isolated from semiarid and seasonally dry environments will be detailed in the present review, focusing on plant growth promotion potential and the microbial ability to alleviate plant abiotic stress. Initially, we explored the role of microbes from dry environments around the world, and then, we focused on seasonally dry Brazilian biomes, the Caatinga and the Cerrado. Cultivable bacteria from semiarid and seasonally dry environments have demonstrated great plant growth promotion traits such as plant hormone production, mobilization of insoluble nutrients, and mechanisms related to plant abiotic stress alleviation. Several of these isolates were able to improve plant growth under stressful conditions commonly present in typical semiarid regions, such as high salinity and drought. Additionally, we highlight the potential of plants highly adapted to seasonal climates from the Caatinga and Cerrado biomes as a suitable pool of microbial inoculants to maintain plant growth under abiotic stress conditions. In general, we point out the potential for the exploitation of new microbial inoculants from plants growing in dry environments to ensure a sustainable increase in agricultural productivity in a future climate change scenario.

## Introduction

Plants can host many microbes in the rhizosphere, endosphere, leaf surfaces, and other tissue compartments, collectively known as the plant microbiome. Many of these plant-associated microbes are known to be able to benefit plant health by positively influencing plant physiology, development, and environmental adaptation (Grover et al., 2011; Mendes et al., 2011; Berendsen et al., 2012). The plant microbial community composition and functions are tightly shaped by environmental factors (Martiny et al., 2006; Taketani et al., 2014, 2017) and changes in root and leaf exudates and other metabolic inputs (Williams and de Vries, 2020). The stress-induced enrichment of particular microbes in different host tissues can mediate plant stress tolerance (Liu et al., 2020).

Arid lands cover approximately 30% of the world’s land area. Particularly in semiarid and seasonally dry regions, drought, high temperature, and high salinity are commonly reported abiotic stresses that have a significant impact on the plant-associated microbiota (Kavamura et al., 2013b; Paul and Lade, 2014; Naylor and Coleman-Derr, 2018; Lacerda-Júnior et al., 2019). Plants and microbes have some adaptations and interactions to allow them to survive in these harsh environments and to overcome such harsh conditions (Ngumbi and Kloepper, 2016). Plant-associated bacteria that positively influence plant growth and development are collectively known as plant growth-promoting bacteria (PGPB). Given the current climate change scenario and future predictions of an increase in arid lands (Beck et al., 2018), the prospect of PGPB from semiarid and seasonally dry regions to enhance crop yield under stress conditions has been explored (Kavamura et al., 2013a; Marasco et al., 2013; Shrivastava and Kumar, 2015; Jochum et al., 2019; Selim et al., 2019; Andrade et al., 2020; Santana et al., 2020; Santos et al., 2020).

The best-known mechanisms involved in plant growth promotion are the production of plant phytohormones [such as indoleacetic acid (IAA) and gibberellins] (Bano et al., 2010; Duca et al., 2014; Singh and Jha, 2017), mobilization of insoluble nutrients (P, Fe, and Zn, for example) (Chen et al., 2006; Chauhan et al., 2017), biological control of soilborne pathogens (Dias et al., 2014), biological nitrogen fixation (BNF) (Baldani et al., 2014), and 1-aminocyclopropane-1-carboxylic acid (ACC) deaminase production (Glick et al., 1998; Glick, 2005). Some plant growth-promotion mechanisms (PGPMs), such as exopolysaccharide production (Bomfeti et al., 2011) and higher accumulation of osmoprotective sugars and amino acids (threalose, proline, and betaine) (Naseem et al., 2018), are more specifically involved in plant tolerance to common abiotic stresses found in dry regions (e.g., drought and salinity).

Thus, this review will initially focus on assessing the potential and features of bacteria from semiarid environments worldwide exhibiting plant growth-promoting (PGP) traits to alleviate abiotic stresses. Additionally, we explored the biological diversity of two seasonally dry environments as a prospective microbial inoculant source to promote important crop growth under stress conditions. The Caatinga, an exclusively Brazilian biome, comprises a highly diverse tropical dry forest that faces a seasonal water regime (de Albuquerque et al., 2012; Alvares et al., 2013). The Cerrado, the largest and most taxon-rich savanna in the world, is considered one of 25 global biodiversity hotspots (Myers et al., 2000). Their extensive natural vegetation highly adapted to harsh conditions may harbor new PGPB with beneficial plant–microbe interactions facing environmental stressors.

## Prospects of Plant Growth-Promoting Bacteria From Semiarid Regions

Plants from semiarid regions are exciting sources of promising PGPB, as they may harbor associated microbes with the potential to alleviate abiotic stress related to dry environments (Shrivastava and Kumar, 2015). For this reason, several studies have focused on isolating and investigating bacteria inhabiting plants adapted to semiarid regions. Regarding the choice of the host as a microbial source, both native plants from semiarid climates (Sorty et al., 2016; El-Kahkahi et al., 2019; Gao et al., 2019) and crop plants (Egamberdieva et al., 2008; Yasmin et al., 2013) grown in semiarid lands have been explored.

Usually, investigation of PGPB from semiarid regions searches for common traits related to direct plant growth promotion such as ACC deaminase activity, nitrogen fixation, phosphate solubilization, IAA production, and siderophore production (Kumar et al., 2014; Wang et al., 2014; Niu et al., 2018; El-Kahkahi et al., 2019; Gao et al., 2019; de la Torre-Hernández et al., 2020). However, some studies have also focused on the indirect mechanism of plant growth, such as the biocontrol potential of bacterial isolates (Egamberdieva et al., 2008; Minaxi and Saxena, 2010; Yasmin et al., 2013; Table 1).

**TABLE 1:** Prospect of plant growth-promoting bacteria from plants growing in semiarid lands and seasonally dry environments and the traits investigated.

Authors	Location	Host Plant	Sample source	Bacterial isolates	PGP traits*
Egamberdieva et al., 2008	Uzbekistan	*Triticum aestivum*	Rhizosphere	*Acinetobacter* sp., *Alcaligenes faecalis*, *Bacillus cereus*, *Enterobacter hormaechei*, *Pantoea agglomerans*, *Pseudomonas aeruginosa*, *Staphylococcus saprophyticus*	Antagonistic activities; Exoenzymatic activities; Indol Acetic Acid (IAA) production; Salt tolerance; Temperature tolerance; Volatile hydrogen cyanide (HCN) production
Fernandes-Júnior et al., 2015; Santana et al., 2020	Brazil	*Tripogonella spicata (Nees)*	Root	*Pantoea* sp., *Bacillus* sp., *Enterobacter* sp., *Rhizobium* sp.	IAA production; nitrogen fixation under saline conditions
Gao et al., 2019**	China	*Caragana microphylla*	Rhizosphere, bulk soil	Actinobacteria, Firmicutes, Proteobacteria	1-aminocyclopropane-1-carboxylate (ACC) deaminase activity; IAA production; nitrogen fixation; phosphate solubilization; Siderophore production
Jochum et al., 2019	United States	*Cynodon* spp.	Rhizosphere	*Bacillus* sp., *Enterobacter* sp.	Drought tolerance; phytohormone production
Kavamura et al., 2013a	Brazil	*Cactus (Cereus jamacaru)*	Root	*Bacillus* spp., *Pantoea* sp., *Virgibacillus* sp., *Brevibacillus* sp., *Arthrobacter* sp., *Paenibacillus* sp., *Gordonia* sp., *Cellulosimicrobium* sp., *Nocardia* sp.	Cellulase production; exopolysaccharide production; growth in medium with low water availability (0.919Aw); HCN production; IAA production; NH_3_ production; phosphate solubilization; salt tolerance
El-Kahkahi et al., 2019	Morocco	*Ceratonia siliqua* L.	Rhizosphere	*Bacillus* spp., *Pseudomonas gessardii*	Cellulase production; chitinase production; IAA production; nitrogen fixation; phosphate solubilization; protease production
Kumar et al., 2014**	India	Not informed	Rhizosphere	*Agrobacterium tumefaciens*, *Bacillus* spp., *Paenibacillus* sp., *Stenotrophomonas* sp.	Acetylene reduction; ammonia excretion; IAA production; phosphate solubilization; siderophore production
Minaxi and Saxena, 2010	India	*Vigna radiata* L.	Rhizosphere	*Burkholderia cepacia*, *Pseudomonas fluorescens*	Antagonistic activities; chitinase production; siderophore production
Niu et al., 2018	China	*Setaria italica* L.	Root	*Arthrobacter* spp., *Enterobacter* spp., *Klebsiella oxytoca*, *Ochrobactrum intermedium*, *Pantoea* spp., *Pseudomonas* spp.	ACC deaminase activity; drought tolerance; exopolysaccharide production; IAA production; nitrogen fixation; phosphate solubilization; siderophore production
Sorty et al., 2016	India	*Psoralea corylifolia* L.	Root, shoot and nodule endophytes, rhizosphere, rhizoplane, leaf epiphytes	*Acinetobacter* sp., *Bacillus* sp., *Enterobacter* sp., *Marinobacterium* sp., *Pantoea* sp., *Pseudomonas* sp. *Rhizobium* sp., *Sinorhizobium* sp.	Exopolysaccharide production; IAA production; nitrogen fixation; phosphate solubilization; salt tolerance; siderophore production
de la Torre-Hernández et al., 2020	Mexico	*Echinocactus platyacanthus*	Rhizosphere	*Bacillus* spp., *Brevibacterium* sp., *Cutibacterium* sp., *Paenibacillus* sp., *Pseudomonas* spp., *Stenotrophomonas* spp.	Antagonistic activities; IAA production; phosphate solubilization; siderophore production
Wang et al., 2014**	China	*Populus euphratica*	Rhizosphere	*Bacillus* sp., *Pseudomonas* sp., *Serratia* sp., *Stenotrophomonas* sp.	ACC deaminase activity; acetoin production; IAA production; nitrogen fixation; phosphate solubilization; siderophore production
Yasmin et al., 2013	Pakistan	*Zea mays*	Rhizosphere	Not informed	Bacteriocin production; phosphate solubilization; siderophore production

*^∗^PGP traits—Plant growth promotion traits. ^∗∗^Samples collected from both arid and semiarid regions.*

Because semiarid areas impose drought and high salinity stresses on plants and the associated bacterial community (Paul and Lade, 2014; Naylor and Coleman-Derr, 2018), strategies to isolate bacteria from plant hosts that can cope with such stresses are desirable. For instance, Niu et al. (2018) isolated bacterial strains from the roots and rhizosphere of foxtail millet (*Setaria italica* L.), a drought-tolerant crop growing in a semiarid agroecosystem in northeastern China, and they were able to find four isolates with the potential to alleviate drought stress. Similarly, Egamberdieva et al. (2008) isolated 210 rhizobacteria from wheat roots grown in salinized soils in a semiarid climate in Uzbekistan, and they found eight isolates that positively affected wheat plant *in vitro* growth and that were salt-tolerant.

The prospect of PGPB to alleviate semiarid stresses could benefit from a more targeted approach. Sorty et al. (2016) isolated 79 bacteria associated with nodule, root, stem, leaf, and root surfaces and rhizosphere of *Psoralea corylifolia* L. grown in a salt-affected semiarid region of India. They found that bacteria isolated from the rhizosphere and rhizoplane showed the highest salt-tolerance ability, probably due to their previous exposure to high salt concentrations in the soil. Such information could guide future studies interested in isolating halotolerant bacteria.

Moreover, in another report, Jochum et al. (2019) designed a novel bioprospecting procedure to screen PGPB capable of rapidly colonizing the rhizosphere and mitigating drought stress in multiple cereal hosts. Initially, they isolated 200 bacteria from the perennial grass rhizosphere of a semiarid environment in Texas, United States. These plants are able to grow vigorously under water stress, so they might foster a microbiome capable of coping with water stress. Then, they conducted a prescreening focusing on the desired host phenotype (onset delay of drought stress symptoms) rather than bacterial phenotype. With such an approach, they were able to find two bacterial isolates that significantly alleviated drought stress symptoms.

In summary, when investigating PGPB from semiarid regions, one should consider: (1) the host choice, evaluating its habitat conditions; (2) prospecting bacterial common PGP traits; (3) prospecting bacterial PGP traits related to dry environment stress alleviation; and (4) using more targeted approaches, focusing either on a particular abiotic stress alleviation or on a specific plant to enhance growth.

## Evaluating the Plant Growth Promotion of Bacterial Isolates From Semiarid Environments

**FIGURE 1:**
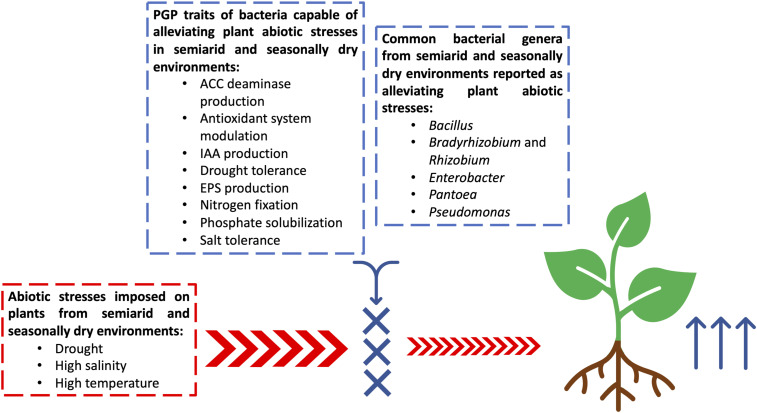
Schematic figure showing plant growth-promoting bacteria (PGPB) from semiarid and seasonally dry environments able to promote drought and salinity stress alleviation and their plant growth-promoting (PGP) traits. Abbreviations: ACC deaminase, 1-aminocyclopropane-1-carboxylate deaminase, IAA, indoleacetic acid, EPS, Exopolysaccharide production.

Bacteria that exhibit several PGP traits—such as nitrogen fixation, phosphate solubilization, phytohormone production, and salt and drought tolerance—become interesting candidates to be evaluated under greenhouse and field conditions aiming for the selection of new plant growth promoters under the harsh environmental conditions of a semiarid climate (Figure 1). Bacterial strains isolated from a particular plant should be tested to promote the growth of the same plant (Arif et al., 2016; Mishra et al., 2016) as well as of an important food crop such as wheat and maize (Wang et al., 2014; Sorty et al., 2016; Jochum et al., 2019; Table 2). The cross-colonization of bacterial isolates is common (Quecine et al., 2012) and has been used to alleviate drought, common abiotic stress in semiarid lands (Marasco et al., 2013).

**TABLE 2:** Plant growth-promoting bacteria from semiarid lands and seasonally dry environments, and their role in alleviating abiotic stresses in crops.

Authors	Location	Host plant	Sample source	Tested plant	Bacterial isolates	Conditions of experiment
AbdElgawad et al., 2019	Saudi Arabia	*Phoenix dactylifera* L.	Rhizosphere	*Phoenix dactylifera* L.	*Actinobacteria* isolates	Field in semiarid climate
Arif et al., 2016	Pakistan	*Helianthus annuus* L.	Rhizosphere	*Helianthus annuus* L.	*Pseudomonas aeruginosa*	Presence or absence of Nitrogen
Armada et al., 2016	Spain	Not informed	Soil	*Lavandula dentata*	*Bacillus thuringiensis*	Drought stress
Jochum et al., 2019	United States	*Cynodon* spp.	Rhizosphere	*Triticum aestivum*, *Zea mays*	*Bacillus* sp., *Enterobacter* sp.	Drought stress
El-Kahkahi et al., 2019	Morocco	*Ceratonia siliqua* L.	Rhizosphere	*Ceratonia siliqua* L.	*Bacillus* spp., *Pseudomonas gessardii*	Field in semi-arid conditions
Kavamura et al., 2013a	Brazil	*Cereus jamacaru* (Cactus)	Rhizosphere	*Zea mays*	*Bacillus* spp. *and Pantoea* sp.	Greenhouse experiment/drought stress
Minaxi et al., 2012	India	*Vigna radiata*	Rhizosphere	*Vigna unguiculata*	*Bacillus* sp.	Field in semiarid conditions
Minaxi and Saxena, 2010	India	*Vigna radiata*	Rhizosphere	*Vigna radiata*	*Burkholderia cepacia*, *Pseudomonas fluorescens*	Biocontrol
Mishra et al., 2016	India	*Foeniculum vulgare Mill.*	Soil	*Foeniculum vulgare* Mill.	*Bacillus* spp., *Planococcus* sp.	Field experiment
Misra et al., 2017	India	Agricultural fields	Soil	*Oryza sativa*	*Acinetobacter lwoffii*, *Arthrobacter defluvii*, *Bacillus* spp., *Jeotgalicoccus huakuii*, *Lysinibacillus fusiformis*, *Oceanobacillus picturae*, *Staphylococcus cohnii*	Salinity stress
Sandhya et al., 2010	India	Crop plants	Rhizosphere	*Zea mays*	*Pseudomonas* spp.	Drought stress
Santana et al., 2020	Brazil	*Tripogonella spicata*	Roots	*Zea mays*	*Enterobacter* sp., *Bacillus* sp., and *Rhizobium* sp.,	Drought stress
Selim et al., 2019	Saudi Arabia	*Panicum turgidum*	Rhizosphere	*Zea mays*	*Streptomyces* sp.	Drought stress
Sorty et al., 2016	India	*Psoralea corylifolia*	Root, shoot and nodule endophytes, rhizosphere, rhizoplane, leaf epiphytes	*Triticum aestivum*	*Acinetobacter* sp., *Bacillus* sp., *Enterobacter* sp., *Marinobacterium* sp., *Pantoea* sp., *Pseudomonas* sp. *Rhizobium* sp., *Sinorhizobium* sp.	Salinity stress
Wang et al., 2014	China	*Populus euphratica*	Rhizosphere	*Triticum aestivum*	*Bacillus* sp., *Pseudomonas* sp., *Serratia* sp., *Stenotrophomonas* sp.	Drought stress

Bacteria able to promote plant growth under drought stress significantly affect root architecture and/or growth, improving water and nutrient acquisition (Wang et al., 2014; Armada et al., 2016; Jochum et al., 2019). The root improvement can be related to the bacterial production of phytohormones (Glick, 1995)—such as IAA—whereas this trait was noticed in bacteria able to promote plant growth under drought conditions (Armada et al., 2016; Jochum et al., 2019; Selim et al., 2019). However, it is essential to point out that root improvement might also be related to other plant–microbe interaction mechanisms.

Oxidative stress, characterized by the enhanced production of various reactive oxygen species (ROS), is one of the main causes of plant injury under drought stress (Hasanuzzaman et al., 2013; Ngumbi and Kloepper, 2016). Plants maintain ROS homeostasis by producing enzymes and antioxidant compounds (Carvalho, 2008). In this context, bacteria capable of reducing harmful plant oxidative effects are also potential candidates to increase drought tolerance. Plants inoculated with PGPB exhibited decreased production of malondialdehyde, a biochemical marker for oxidative lipid injury induced during drought (Armada et al., 2016; Selim et al., 2019).

Previous reports have shown an increase in antioxidant enzymes in plants inoculated with PGPB under drought conditions (Ngumbi and Kloepper, 2016). However, plants inoculated with bacterial isolates from semiarid regions have shown different trends relative to antioxidant system activity.

The antioxidant activities of superoxide dismutase, catalase, and ascorbate peroxidase enzymes in *Lavandula dentata* depend on the single or co-inoculation of different arbuscular mycorrhizal fungi with endophytic *Bacillus thuringiensis.* In particular, the interaction of the arbuscular mycorrhizal fungal mixture and *B. thuringiensis* induced the highest activity of some antioxidant plant enzymes and increased mycorrhizal development (Armada et al., 2016). Likewise, actinobacteria also increased the level of molecular antioxidants (total ascorbate, glutathione, tocopherols, phenolic acids, and flavonoids) and improved the growth and photosynthesis of maize grown under water-deficit conditions (Selim et al., 2019). In contrast, maize seedlings inoculated with *Pseudomonas* spp. showed lower antioxidant enzyme activities (ascorbate peroxidase, catalase, and glutathione peroxidase) under drought stress. The reduced drought stress effect on the antioxidant activity in inoculated seedlings may be explained by the protective effect granted by other bacterial traits that avoid dehydration (Sandhya et al., 2010). Clearly, the plant antioxidant system is essential to control the damage of ROS; however, more studies are necessary to support the mechanisms behind plant oxidative stress alleviation under drought conditions by PGPB.

Salt stress is another important abiotic stress limiting the productivity of crop plants in arid and semiarid conditions. Saline soils increase the levels of ethylene and ACC, causing plant damage (Kukreja et al., 2005). Interestingly, the inoculation of ACC deaminase-producing bacteria can help plants cope with salinity by reducing ethylene levels (Paul and Lade, 2014).

Indigenous bacteria of nine agroclimatic zones from India were selected based on their salt tolerance ability and ACC deaminase activity. Eighty-eight percent of isolates from the semiarid region were salt-tolerant, and the isolate *Bacillus megaterium* NBRI 20M exhibiting the highest ACC deaminase activity was capable of alleviating maximum salinity stress in rice (Misra et al., 2017). However, it is important to point out that other isolates with high production of IAA and low activity of ACC deaminase were responsible for significant increases in rice seedling biomass (Misra et al., 2017). These findings make it clear that despite the proven growth promotion mediated by ACC deaminase activity in decreasing ethylene levels (Glick, 2005), it is not easy to disentangle the actions and effects of each mechanism when using multitrait PGPB.

Plant growth promotion by multitrait PGPB can occur through the action of more than one mechanism at a time. In our study, several bacterial isolates from semiarid regions showed some PGP traits (Minaxi et al., 2012; Armada et al., 2016; Selim et al., 2019). It is also common to look for more than one trait when prospecting for potential PGPB (see Table 1). However, more studies should focus on understanding the mechanisms used independently by bacteria to alleviate abiotic stresses. One interesting approach that might be used to investigate the molecular mechanisms related to plant growth promotion in bacteria is the knockout of a gene involved in the metabolism of such traits. This approach has been successfully used to investigate genes related to IAA metabolism (Idris et al., 2007; Malhotra and Srivastava, 2008) and ACC deaminase (Li et al., 2000).

Finally, when looking for a successful PGPB, field experiments are an important approach to evaluate how they will perform under real conditions. Several bacterial isolates were able to promote plant growth of some crops under semiarid field conditions. *Bacillus* sp. RM-2 enhanced the yield parameters of cowpea plants (number of pods and seeds and grain yield), showing several PGP traits (Minaxi et al., 2012). Actinobacterial inoculants were able to increase soil fertility and improve fruit yield in date palm plants. The fruits showed higher levels of valuable phytochemicals, suggesting that inoculation improves production and functional food value (AbdElgawad et al., 2019). Additionally, the *Bacillus* and *Pseudomonas* genera promoted carob tree plant growth by enhancing plant height, root length, and fresh weight of the aerial and root parts in a field experiment (El-Kahkahi et al., 2019).

## Brazilian Semiarid Region and Seasonally Dry Environments: A Promising Source of Potential Plant Growth-Promoting Bacteria

**FIGURE 2:**
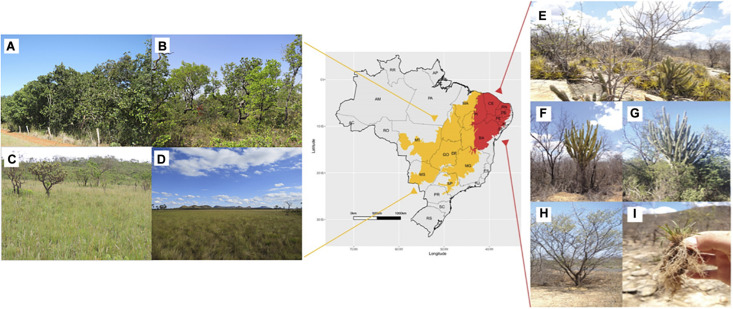
Map represents the Caatinga (red) and Cerrado (yellow) biomes in Brazil. Main phytophysiognomies of Cerrado savanna are shown on the left (Ribeiro et al., 1983; Ribeiro and Walter, 2008). **(A)** Dense cerrado or “cerradão” has a tree cover from 50 to 90%. This phytophysiognomy corresponds to a forest formation composed of small shrubs and herbs with few kinds of grass. **(B)** “Cerrado” *sensu stricto* (tree cover of 20–50%) harbors small and tortuous trees with irregular and twisted branches. In the rainy season, the sub-shrub and herbaceous strata are exuberant due to their rapid growth. **(C)** Cerrado grassland or “campo sujo” is an exclusively shrub-herbaceous physiognomic type. **(D)** Grassland or “campo limpo” is a predominantly herbaceous phytophysiognomy, with rare shrubs and a complete absence of trees (Pictures were taken and provided by Dr. Marcelo Simon—EMBRAPA Genetic Resources and Biotechnology). **(E)** Overview of the tropical Caatinga dry forest landscape showing the trees and shrubs highly adapted to the semiarid climate in Petrolina, PE, Brazil. **(F,G)** Pictures of *Cereus jamacaru*, a native cactus widely distributed in the Caatinga biome, during the dry and rainy seasons, respectively. **(H)** “Jurema-preta” (*Mimosa tenuiflora*). **(I)** *Tripogonella spicata* (Ness), a desiccation-tolerant grass from hyperxerophic regions of the Caatinga biome.

**FIGURE 3:**
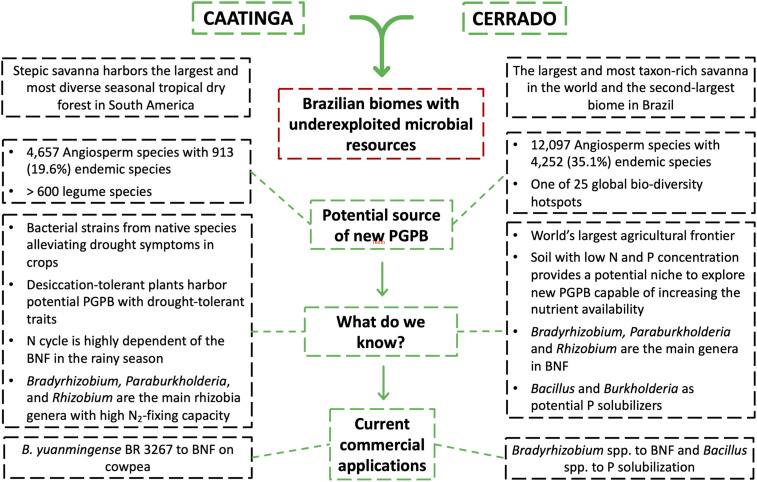
Schematic figure illustrating the main characteristics and the potential of the Brazilian Cerrado and Caatinga biomes as a promising source for prospecting for new plant growth-promoting bacteria (PGPB) with commercial application in agriculture. Abbreviation: BNF, biological nitrogen fixation.

Tropical countries housing peculiar ecosystems evolving under relatively harsh environmental conditions are expected to harbor unique and diverse biota and remarkable microbes. Especially in Brazil, the Caatinga tropical dry forest and Cerrado savanna are dryland ecosystems with a high diversity of native plant species adapted to thrive in a seasonally variable climate, high temperatures, high levels of ultraviolet radiation and salinity, and low-nutrient soils (Damasco et al., 2018; Figure 2). Due to their high biological diversity even under peculiar climate conditions, these Brazilian ecosystems may be explored to decipher plant–microbiome interactions to confront abiotic stresses and identify potential new PGPBs (Figure 3).

## Plant-Associated Bacteria From the Caatinga Biome and Their Plant Growth-Promoting Potential Under Drought Stress

As shown in the previous sections, the beneficial microbes naturally associated with plants growing in semiarid environments have attracted increased interest. They represent an unexploited reservoir for biological agents against biotic stresses that endanger agricultural ecosystems. The Caatinga is a stepic savanna found exclusively in the heart of the Brazilian semiarid belt in the northeast region (Figure 2). The Caatinga biome harbors the largest and most diverse seasonal tropical dry forest in South America, covering 844,453.00 km^2^ (approximately 11% of the Brazilian territory) (Beuchle et al., 2015; Queiroz et al., 2017). Its general ecophysiognomy presents a heterogeneous mosaic of drought-tolerant plants: succulents, thorny deciduous trees, and shrubs with xerophytic characteristics (Figure 2). The set of contrasting physical and climatic factors provides a high diversity of vegetation types. In this huge extension, Caatinga harbors (according to our current knowledge) 4,657 angiosperm species, of which 913 (19.6%) are endemic (Zappi et al., 2015). During the dry season, most plant species lose their leaves, and white tree trunks prevail in the landscape (Salgado et al., 2015).

Although plants inhabiting the Caatinga semiarid lands possess inherent traits for survival in a harsh climate, the plant-associated microbiome has also been suggested to play an essential role in plant fitness under seasonal water availability and extended drought periods. This point is a neglected topic in plant microbiology, but some recent research has revealed the potential of bacteria associated with Caatinga native plants as inoculants for plant growth promotion under drought conditions.

Water availability is known to be able to shape the microbial community composition, both in bulk soil (Lacerda-Júnior et al., 2019) and the rhizosphere of Caatinga native species (Kavamura et al., 2013b). Using culture-independent analyses, Kavamura et al. (2013b) showed the rhizosphere microbiome seasonal pattern from *Cereus jamacaru* (Figures 2F,G), a well-distributed native cactus in the Caatinga biome. The phyla Actinobacteria and Firmicutes (mainly the *Bacillus* genus) were significantly enriched during the dry season, whereas the phyla Proteobacteria and Bacteroidetes were more abundant in the rainy season. However, although shifts in the rhizosphere microbial community driven by seasonality are also evidenced in other rhizosphere systems (Ibekwe et al., 2017; Liang et al., 2019), their functional role in supporting plant development under arid and semiarid ecosystems is unclear.

One of the hypotheses is that root-associated microorganisms have developed cellular mechanisms for water stress tolerance that lead to plant protection against the negative effects of desiccation (Naseem et al., 2018; Naylor and Coleman-Derr, 2018). To test this hypothesis, Kavamura et al. (2013a) investigated for the first time the potential of cacti root-associated bacteria from Brazilian semiarid lands to promote maize growth (*Zea mays*) under water stress. Most cactus isolates showed *in vitro* PGP traits, exopolysaccharide production, and the ability to grow in a medium with reduced water availability (0.919 Aw) (Tables 1, 2). The inoculation of two xerotolerant cactus-associated *Bacillus* spp. (LMA52 and LMA3) and *Pantoea* sp. in maize seedlings protected against the negative effects of drought and increased the leaf area, stalk length, and shoot dry biomass under water stress. These results indicate that selecting osmotolerant microorganisms with common PGP traits is a promising approach to prospecting potential PGPB to mitigate plant drought stress. More recently, other *Bacillus* spp. from the cactus rhizosphere of Brazilian Caatinga showed the potential to promote maize growth under drought conditions (Santos et al., 2020). These reports reveal cacti as an underexploited source of PGPB to crops in agricultural practices under drought conditions.

Regarding the plant mechanisms to cope with water-limited conditions, desiccation-tolerant (DT) plants or “resurrection plants” can desiccate and survive during severe and long drought periods (Tuba and Lichtenthaler, 2011). *Tripogonella spicata* (Nees) P.M. Peterson and Romasch (Figure 2), a DT wild grass naturally found at rock outcrops in hyperxerophilic regions of the Caatinga biome (Gaff, 1987), possesses specialized physiologic strategies to recover active metabolism after long drought periods (Aidar et al., 2017). However, the role of microbes in helping resurrection plants tolerate and recover photosynthetic activity after long dehydration periods is still not well understood. Then, Fernandes-Júnior et al. (2015) isolated and tested the potential of diazotrophic PGPB from *T. spicata* roots to promote plant growth. Five isolates were able to increase rice root and/or shoot growth compared with plants inoculated with *Azospirillum brasilense* Ab-V5 (Hungria et al., 2010), a commercial strain of rice inoculants in Brazil. Two isolates identified as *Pantoea* sp. and *Bacillus* sp. increased rice growth at the same rate as plants that received full N fertilization. Furthermore, Santana et al. (2020) showed that *Enterobacter* sp., *Bacillus* sp., and *Rhizobium* sp., isolated from *T. spicata*, were also able to improve some plant growth and gas exchange parameters in sorghum growing under drought conditions (Tables 1, 2). Although these bacterial groups have already been shown to be efficient inoculants for sorghum (da Silva et al., 2018; Antunes et al., 2019), their ability to attenuate the adverse effects of drought and promote sorghum growth under drought had not been revealed. Interestingly, these *T. spicata* isolates showed diazotrophic capacity in a culture medium supplemented with up to 1.27 mol L^–1^ NaCl (Table 1; Fernandes-Júnior et al., 2015), which suggests that the beneficial effects of BNF may be supported around the plant environment under water-deficit conditions. These results indicate that the selection of strains able to show active PGP traits under salt and water stress conditions may be a rational strategy to identify beneficial microbes to help plants cope with harsh environments. In contrast to microbes thriving in common lands, the plant-associated microbiome from dry ecosystems has been selected based on phenotypic or genomic attributes related to a more rigorous stress response regulation of their hosts.

The biological process related to vegetative desiccation tolerance is exhibited by less than 0.2% of vascular plant species. In addition to *T. spicata*, several other DT plants, such as the pteridophyte *Selaginella* spp. (Selaginellaceae), *Vellozia cenerascens* (Velloziaceae), and *Nanuza plicata* (Velloziaceae), are found in the Caatinga biome. These data show that plants from Caatinga semiarid areas display a promising niche for isolating novel beneficial microbes with remarkable plant growth-promoting rhizobacteria traits. Research efforts to study the indigenous microbes associated with these species open possibilities for prospecting for new chemical and biological agents to improve the growth of different crop species in dry agricultural lands.

## Legume–Rhizobia Associations in the Caatinga Biome: Diversity and Their Potential Exploration

Legume plants are the unique family with the ability to establish mutualistic associations with “rhizobia,” a collective term given to root/stem-associated bacteria that form nodule structures to the BNF. All rhizobia are members of the phylum Proteobacteria within the classes α- and β-proteobacteria, also known as α- and β-rhizobia (Peix et al., 2015). A recent review by Wang (2019) listed 190 species of rhizobia divided into 21 nodulating genera. However, this number is continuously increasing, mainly because new rhizobial species are frequently described.

There is a lack of knowledge regarding the diversity of indigenous rhizobia from native legume species of the Caatinga biome. However, studies evaluating the rhizobial diversity in Caatinga native plant roots have been published in the last few years, indicating the existence of potential new taxa. Genetic analyses of the rhizobial culture collections isolated from *Mimosa* spp. through amplified ribosomal DNA restriction techniques reveal high diversity and low similarities with several known reference isolates (Teixeira et al., 2010; de Freitas et al., 2014). Likewise, Martins et al. (2015) showed that *Mimosa caesalpinifolia* nodulates primarily with *Paraburkholderia* spp. in pristine Caatinga areas. Interestingly, the 16S ribosomal RNA gene analyses indicated that some of them were not closely related to the reference strains available in the GenBank database.

From the same legume, de Oliveira et al. (2019) compared the taxonomic diversity of *Paraburkholderia* from different Brazilian regions using the multilocus sequence analysis approach. The bacterial members isolated from the semiarid region in the Caatinga biome diverged from those in other biomes. β-Rhizobia from drylands were related to *Paraburkholderia diazotrophica* or *Trinickia symbiotica*, whereas bacteria isolated from Rio de Janeiro state (far from the Brazilian drylands) were closely related to the *Paraburkholderia sabiae* and *Rhizobium* genera. Furthermore, rhizobial isolates from Caatinga drylands were more efficient in increasing plant biomass in gnotobiotic inoculation assays. These results corroborate dos Reis Junior et al. (2010), who described, for the first time, the nodulation of *Mimosa* spp. by *Paraburkholderia* (Syn. *Burkholderia*) in Caatinga and Cerrado Brazilian biomes. A large diversity of *Paraburkholderia* also nodulate other Mimosoid legumes. In the case of *Calliandra*, although the majority of the β-rhizobia were related to *Paraburkholderia nodosa*, some isolates were not related to known rhizobia from *Mimosa* hosts (Silva et al., 2019). Bournaud et al. (2013) also isolated both β- and α-rhizobia in the genera *Paraburkholderia* and *Rhizobium*, respectively, from *Piptadenia viridiflora* growing in the Caatinga biome. These results point to the large diversity of the nodulation patterns of mimosoid legumes from Brazilian drylands, showing vast potential to be explored in the search for new PGPB.

The diversity and ecology of rhizobial papilionoid native legumes in Caatinga are poorly understood. In recent years, bacterial isolation efforts have shown the high biodiversity and biotechnological potential of some rhizobial species. *Erythrina velutina*, also known as “mulungu,” is an iconic Brazilian tree with several potential applications for sustainable use, such as ethnobotanical and traditional medical applications. Regarding the production of healthy mulungu seedlings, the selection of efficient rhizobial isolates is desirable. Studies assessing the diversity and symbiotic potential of mulungu rhizobia indicated that *E. velutina* nodulates mainly with α- (*Rhizobium* spp. and *Bradyrhizobium* spp.) rather than β-rhizobia (*Paraburkholderia* spp.), but both classes are found in mulungu nodules (Menezes et al., 2016, 2017; Rodrigues et al., 2018). Considering the symbiotic efficiency, the genera *Rhizobium* and *Bradyrhizobium* encompass promising bacteria with superior symbiotic efficiency compared with the other native isolates and non-inoculated treatments (both with sterile and non-sterile substrates).

Compared with the Papilionoideae and Mimosoideae clades, the Caesalpinoideae clade has fewer nodulating members, comprising approximately 25% of the members within this group (Lewis et al., 2005). For this reason, it is more challenging to find nodulating Caesalpinioideae than Papilionoideae and Mimosoideae in pristine areas. In Caatinga, there is little information about the diversity and efficiency of rhizobia from the Caesalpinioideae family. In a recent survey, dos Santos et al. (2017) isolated several rhizobial isolates from *Chamaecrista* spp. collected in several areas of Bahia state in the heart of Brazilian drylands. The molecular evaluation based on 16S ribosomal RNA, housekeeping, and symbiosis gene sequencing indicated that they belonged to the *Bradyrhizobium* genus. The taxonomic positioning based on the multilocus sequence analysis approach showed that most isolates (12 of 16 bacteria) were clustered in a separated clade that was not related to classical *Bradyrhizobium elkanii* and *Bradyrhizobium japonicum* taxonomic superclades. Overall, these results indicate that diverse rhizobia can nodulate different legumes, even those of closely related species.

Given that the Caatinga biome has (until now) more than 600 legume species, its expected rhizobial diversity is still not revealed. Efforts to isolate and identify these bacteria will probably lead to the description of several new taxa and exploration of potential PGPB traits.

In agricultural systems, several legumes are cropped in the Brazilian semiarid belt (the agricultural lands initially covered by the Caatinga biome), mainly in low-income family based production systems. Among these legumes, cowpea (*Vigna unguiculata*) is one of the most used, being an important crop in the economy, culinariness, and culture of the people living in the Brazilian semiarid region. For cowpea, there are four *Bradyrhizobium* spp. strains used for commercial inoculant production in Brazil. Among these strains, *Bradyrhizobium yuanmingense* BR 3267 was officially recommended in 2004 and is currently the most commonly used in Brazil (da Silva et al., 2012). This strain was isolated from a cowpea-producing field in Petrolina, Pernambuco state, in the heart of the Brazilian dryland (Martins et al., 2003) is highly competitive for nodulation sites, tolerant to abiotic stresses, and shows the ability to fix high amounts of nitrogen, either under rain-fed or irrigated conditions in the Brazilian semiarid region (Martins et al., 2003; Marinho et al., 2014). Along with several promising studies on selecting PGP microbes in the Caatinga biome, the commercial use of the native strain *B. yuanmingense* BR 3267 shows the potential of the Brazilian semiarid region as a source of efficient microbes for agricultural uses.

## Field Assessment of Biological Nitrogen Fixation in Pristine and Recovered Caatinga Areas

It is a challenge to associate the diversity of rhizobia associated with a given tree species and the efficiency of fixing N under field conditions. Among the methodological approaches used to assess the N fixation of native legumes in the Caatinga biome, we can highlight the natural abundance of ^15^N. Briefly, this approach quantifies, using mass spectrometry, the amounts of ^15^N and ^14^N in N_2_-fixing and N_2_-non-fixing species, whereas this difference is used to calculate the total N derived from the atmosphere (fixed) in target species. Then, together with the plant biomass and total N accumulation, the N incorporated in the system (in kg N ha^–1^, for example) is estimated (Boddey et al., 2000).

Teixeira et al. (2006) published the first report of BNF quantification in Brazilian Caatinga fields. The authors investigated the contribution of BNF to N nutrition in *Cratylia mollis*. The results indicated that in the rainy season, *C. mollis* presented up to 86% of N derived from the atmosphere, whereas in the dry season, the amount of fixed N reached 27%, indicating an apparent seasonal effect on the BNF efficiency.

de Freitas et al. (2010) and de Souza et al. (2012) estimated that the contribution of BNF in the three legumes (*Mimosa tenuiflora*, *Mimosa arenosa*, and *Piptadenia stipulacea*) ranged from 6 to 11 kg N ha^–1^ year^–1^ in pristine Caatinga areas. These low amounts were associated with a low density of N_2_-fixing species. In contrast, the authors verified a more significant contribution of N_2_-fixing legumes in Caatinga areas under natural recovery (amount of 130 kg fixed N ha^–1^ year^–1^) due to the high density of fixing legumes, mainly *M. tenuiflora*. These results indicate the contribution of rhizobia–legume associations to nitrogen incorporation in natural systems in semiarid Brazilian areas, even with low successional stages.

Furthermore, soil parameters and atmospheric temperature also influence the total amount of N fixed in Caatinga drylands. The total amount of phosphorus in soils is significantly correlated with the N derived from the atmosphere (biologically assimilated) in pristine and recovering areas (da Silva et al., 2017). This information is important to soil management projects aimed at the recovery of degraded dryland areas. Similarly, de Freitas et al. (2015) showed high N fixation in areas with higher temperatures than the colder regions from the Brazilian Caatinga. Rainfall did not influence the isotopic signals, indicating the positive influence of temperature rather than rainfall on the N fixation process.

Such studies are essential because Caatinga biome degradation is an ongoing problem, and the use of environmentally friendly solutions is needed. Further research should focus on soil management approaches that consider both microbiota and plant diversity to increase fundamental nutrient availability.

## Brazilian Cerrado: A Tropical Savanna Hotspot

The Cerrado, the second largest biome of the Brazilian territory, covers approximately 2 million km^2^ (Figure 2) and is considered the largest and most taxon-rich savannah in the world (Furley, 1999; Myers et al., 2000). The Cerrado harbors approximately 4,200 endemic plant species (Zappi et al., 2015) and a wide range of animals and microorganisms and is considered one of 25 global biodiversity hotspots (Myers et al., 2000). This biome comprises 11 different types of phytophysiognomies, including forests, savannas (shrublands), and grasslands (Ribeiro and Walter, 1998). The typical phytophysiognomy, the Cerrado “sensu stricto,” is composed of a mix of grasses, bushes, and small trees with twisted trunks (Mendes et al., 2012; Figure 2). According to the extensive flora diversity estimated by Zappi et al. (2015) in the Brazilian Cerrado, the degree of endemism is higher than in the Amazon rainforest, comprising 12,097 angiosperm species with 4,252 (35.1%) endemic. The main families are Asteraceae (1,216 species), Fabaceae (1,207), Orchidaceae (727), Poaceae (648), Melastomataceae (484), Eriocaulaceae (461), Rubiaceae (406), Euphorbiaceae (386), Malvaceae (334), and Apocynaceae (293) (Zappi et al., 2015).

The climate of the Cerrado biome is characterized by high-temperature averages (22–27°C), rainfall (800–1,600 mm), and solar radiation (475–500 Cal cm^–2^ day^–1^) (Adamoli et al., 1986; Ratter et al., 1997). Despite the high amount of rainfall, the Cerrado presents a great seasonal contrast between the dry season and the rainy season. The seasonal climate has a strong influence on vegetation phenology. During the dry season, some plants lose all or part of their leaves, increasing the soil biomass and biogeochemical processes in the rainy season (Klink and Solbrig, 1996; Ratter et al., 1997).

Cerrado soils are highly weathered, acidic, deficient in nutrients, and rich in iron and aluminum oxides, supporting only adapted vegetation (Adamoli et al., 1986; Ruggiero et al., 2002). Consequently, nutrient cycling in the Cerrado is a key process regulating ecosystem development and primary productivity (Bustamante et al., 2004). The peculiar soil characteristics such as smooth topography, well-drained, not prone to crusting or compaction, associated with the technological advances in agriculture in the last 40 years, have made the Cerrado one of the world’s largest agricultural frontiers and the leading grain production area in Brazil (Machado and Silva, 2001). However, a high proportion of this biome has been converted into pasture and agricultural fields (Marris, 2005). Land-use mapping revealed that remnant natural vegetation covered approximately 54% of the Cerrado in 2017 (Strassburg et al., 2017).

In addition to the tremendous ecological and economic importance of this biome, little is currently known about its biodiversity, especially its microbial communities. The conversion of natural to agricultural areas in the Cerrado can affect the soil microbial communities and their associated biological processes as a result of changes in soil structure, water content, temperature fluctuations, organic matter and nutrient contents, pH, introduction of plant species, and agrochemical inputs (Castro et al., 2008, 2016; Quirino et al., 2009; Araujo et al., 2012; Souza et al., 2016; de Araujo et al., 2017a, b). Metagenomic analysis showed that microbial communities in agricultural soils have the genetic potential to degrade available carbon and aromatic compounds and the metabolism of N, P, and S, probably in response to the use of fertilizers. In contrast, soils under native vegetation showed a higher number of sequences related to dormancy and sporulation and unknown functions, indicating the possibility of finding new functions and genes (Souza et al., 2016). Another interesting finding from this study was the high number of sequences related to the genus *Bradyrhizobium* in the agricultural soils, in contrast to the higher abundance of *Rhizobium* in the undisturbed Cerrado. The presence of *Rhizobium* species that are tolerant of acidity and other stressful environmental conditions indicates adaptation to the particular edaphoclimatic characteristics of this biome (Ribeiro et al., 2012).

In this context, the peculiar Cerrado soil characteristics combined with high plant diversity provide a potential niche to explore new PGP microbes, especially those capable of increasing availability and nutrient use efficiency, particularly N and P.

## Biological Nitrogen Fixation in Brazilian Cerrado Lands

Tropical savannas are N-limited systems, and the N-cycle depends on the inputs from BNF. A peculiar characteristic of these ecosystems is the high diversity of herbaceous and woody leguminous species associated with native microbiota, including rhizobial strains. More than 1,200 Fabaceae species were documented within the 11 different types of phytophysiognomies in the Brazilian Cerrado. Due to this high diversity, the rhizobial diversity in this biome is expected to be very high.

*Mimosa* is the most diverse genus of Fabaceae in Brazil and one of the most studied regarding the association of Cerrado native plants with rhizobia. Indeed, *Mimosa* is now used as a model for studies involving these symbiotic relationships in natural ecosystems. Studies carried out with these plants have demonstrated that β-proteobacteria (β-rhizobia) play a key role in nitrogen fixation in association with leguminous plants (Pires et al., 2018). Previous studies have shown that bacteria from the genus *Paraburkholderia* (formerly *Burkholderia*) are the primary symbionts of *Mimosa* species in the Cerrado (Elliott et al., 2007; Bontemps et al., 2010; dos Reis Junior et al., 2010). Pires et al. (2018) indicate that soil factors such as pH, nitrogen, organic matter, and fertility seem to prevail over the host identity, determining the predominance of certain types of rhizobia (α- and β-proteobacteria), influencing the establishment of symbiotic relationships.

Dall’Agnol et al. (2016) showed that *P. nodosa* was the main N_2_-fixing species trapped by promiscuous common bean (*Phaseolus vulgaris* L.) in the Brazilian “Cerradão.” “Cerradão” is a type of Cerrado phytophysiognomy that is a forest formation with xeromorphic aspects, and despite the forest-like vegetation, the floristic composition is similar to the Cerrado *sensu stricto* (Figure 2). The predominance of *P. nodosa* might be associated with the edaphic properties of the Cerrado biome, which is characterized by acidic soils with high Al saturation and low nutrient content (Dall’Agnol et al., 2016). Mercante et al. (2017) used Leucaena (*Leucaena leucocephala*) and common bean (*Phaseolus vulgaris* L.) to trap nodules for further rhizobial isolation using Cerrado soils from different locations. This work resulted in the identification of several highly symbiotically efficient *Rhizobium* spp. strains with competitive ability and genetic stability. Common bean inoculation with these strains showed economic viability and high potential to obtain a more effective and suitable inoculant for commercial purposes.

The exploration of the amazing diversity and native and/or naturalized microorganisms adapted to Cerrado edaphoclimatic conditions is a key strategy for the development of highly efficient inoculants to be used in agriculture. An outstanding example is the case of soybean crops, in which new efficient and adapted *Bradyrhizobium* strains have been selected and isolated from areas previously inoculated, showing greater N_2_ fixation capacity, higher competitiveness, and tolerance to frequently stressful tropical conditions (Hungria and Mendes, 2015). Using this approach, it was possible to isolate and select the strains CPAC 7 (*Bradyrhizobium diazoefficiens*) and CPAC 15 (*B. japonicum*), included in the list of strains authorized for use as soybean commercial inoculants in 1992. These strains are currently used successfully by farmers in the Cerrado region. It is important to note that the annual economic return of BNF with soybeans in Brazil is estimated to be approximately US$ 15 billion (Hungria and Mendes, 2015).

## Cerrado Soils as a Source of Phosphate-Solubilizing Microbes

Phosphorus limitation is another challenge to plant growth in Cerrado soils. The strategy of increasing P fertility with corrective fertilizer amendments is economically and environmentally limited due to the high amounts required and the low efficiency in response to the P-fixing capacity of these soils. To overcome these issues, microorganisms able to solubilize and mineralize P from inorganic and organic pools of total soil P have been used as inoculants to enhance plant P availability and acquisition. Oliveira et al. (2009) carried out a study to isolate and evaluate the phosphate solubilization activity of microorganisms associated with maize grown in Cerrado soil and select potential microbial inoculants. *Bacillus* sp. and *Burkholderia* sp. showed the greatest solubilization in media containing tricalcium phosphate, whereas the fungi *Aspergillus terreus*, *Talaromyces rotundus*, and *Penicillium citrinum* were the most effective in solubilizing P sources from aluminum, phytate, and lecithin, respectively. Abreu et al. (2017) reported for the first time the characterization of endophytic bacteria from maize growing in Cerrado lands and their potential to improve crop plant P acquisition in tropical soils. As a result of this work, the first Brazilian inoculant capable of increasing maize absorption of phosphorus was recently released (Embrapa, 2019). This product contains strains of *Bacillus subtilis* (isolated from maize leaf endosphere) and *B. megaterium* (isolated from maize rhizosphere), with the capacity to induce higher maize yield and grain P content under field conditions (de Sousa et al., 2020).

The high microbial diversity from Cerrado soils is expected to present a diverse number of features that could be explored biotechnologically. For example, Braga et al. (2018) isolated *Bacillus* spp. from the rice rhizosphere in the Cerrado, showing positive activity for catalase, protease, amylase, and nitrogenase; antibiosis against the phytopathogens *Rhizoctonia solani* and *Sclerotinia sclerotiorum*; phosphate solubilization; and improved rice plant growth under greenhouse conditions. Carrim et al. (2006) isolated bacteria from the leaves and stems of *Jacaranda decurrens* Cham (Bignoniaceae) and screened for some enzymes of biotechnological interest. The isolates of *Bacillus* spp., *Pseudomonas* spp., *Corynebacterium* spp., *Actinomyces* spp., and *Staphylococcus* spp. presented differential proteolytic, amylolytic, lipolytic, and esteratic activities. These studies show the vast potential of microorganisms associated with Cerrado plants for agricultural improvement and highlight the increasing expectations about the future use of these biological assets.

## Conclusion

These results collectively show that assessing the plant-associated microbial diversity from semiarid regions could be an essential strategy to find PGPB that can successfully alleviate plant abiotic stress commonly present in such climates. Additionally, their plant growth promotion potential has become an interesting alternative to enhance crop yield using more sustainable agricultural practices. With society’s eyes focusing on sustainable exploitation of natural resources and life quality improvement with environmentally friendly technologies, efforts to discover new PGPBs from harsh environments may sustain agricultural productivity in a future scenario of climate change and increased arid lands.

## Author Contributions

MB, GL-J, IM, and MQ contributed to the conception of this review. MB and MQ analyzed the plant-associated microbial diversity in semi-arid regions around the world. GL-J, FRJ, PF-J, and IM focused on the diversity of plant-assocated microbes from Brazilian semiarid and seasonally dry environments. MB and GL-J contributed equally to this work and have combined the first authorship. All authors contributed to manuscript revision and read and approved the submitted version.

## Conflict of Interest

The authors declare that the research was conducted in the absence of any commercial or financial relationships that could be construed as a potential conflict of interest.
